# Why science must speak differently

**DOI:** 10.1186/s13027-025-00707-6

**Published:** 2025-10-30

**Authors:** Francesco Branda, Laura Leondina Campanozzi, Fabio Scarpa, Vittoradolfo Tambone, Massimo Ciccozzi

**Affiliations:** 1 Unit of Medical Statistics and Molecular Epidemiology, Università Campus Bio-Medico di Roma, 00128 Rome, Italy; 2 Genomics, AI, Bioinformatics, Infectious Diseases, Epidemiology Group (GABIE), Rome, Italy; 3 Research Unit of Bioethics and Humanities, Department of Medicine and Surgery, Università Campus Bio-Medico di Roma, 00128 Rome, Italy; 4 Department of Biomedical Sciences, University of Sassari, 07100 Sassari, Italy

**Keywords:** Science communication, Public trust in science, Infodemic, Infocracy, Epistemic humility, Health crises, Uncertainty, Persuasive communication

## Abstract

The COVID-19 pandemic highlighted the complexities surrounding public trust in science, particularly in the context of overwhelming data and political polarization. The call to “trust the science” emerged as both a symbol of confidence and a source of public tension, exposing the challenges in communicating scientific uncertainty, data interpretation, and expertise. This paper examines the critical role of communication in shaping public perceptions of science, emphasizing the need for epistemic humility and transparency in the face of uncertainty. While data availability increased, the real challenge lay in its interpretation and the framing of scientific messages for diverse audiences. The paper argues that health crises, such as COVID-19 and the resurgence of West Nile Virus, demonstrate that information overload and poor communication can lead to confusion, mistrust, and the politicization of science. Effective science communication must transcend purely rational approaches and address emotional and social factors through persuasive strategies, including emotional appeals and interactive tools. Moreover, the structural transformation of information flows in digital societies, epitomized by the concept of “infocracy”, exacerbates the challenge of maintaining public trust in science. The paper calls for a reimagined approach to science communication that prioritizes clarity, context, and responsible engagement, fostering a more informed, resilient, and critically engaged public.

In the wake of the COVID-19 pandemic, the call to “trust the science” emerged as both a rallying cry and a point of contention. This expression, which gained widespread use in institutional campaigns and political discourse, has been critically examined in recent literature as an emblem of both confidence in scientific knowledge and frustration with uncertainty and politicalization in science [1–4]. Its dual function underscores the intricacy of public attitudes towards expertise, particularly in instances where science intersects with policy, values, and emotion. As societies grappled with the challenge of processing enormous quantities of scientific data, public trust in science became intricately intertwined with concerns regarding communication, expertise, and the validation of knowledge.

The public’s trust in science, particularly during health emergencies, is often contingent on the manner in which uncertainty, data, and expertise are communicated. Trust is not merely a byproduct of knowledge dissemination; rather, it is actively shaped by institutional transparency, communicative context, and the perceived credibility of experts [5, 6]. The tensions that surfaced during the course of the pandemic have re-emerged in more recent contexts, such as the management of the West Nile Virus (WNV), thereby underscoring enduring challenges in scientific authority and public understanding. The crux of this issue pertains to the following questions about how science is mediated, who is authorized to speak, and how audiences interpret what they hear. These themes remain pivotal to contemporary debates on credibility, responsibility, and the ethics of communication.

Health crises such as COVID-19 and, more recently, the resurgence of WNV have demonstrated that access to data does not guarantee understanding. During the pandemic, many societies encountered a surfeit of data that lacked contextual significance. Indicators such as the R_t_ index, which measures the average number of secondary infections caused by a single case, became widespread in public discourse. However, such indicators were often misunderstood and inconsistently reported. The challenge was not the availability of data, but rather the interpretation, framing and communication of that data to diverse audiences. In a multitude of instances, scientific uncertainty has been erroneously interpreted as either contradiction or incompetence. The communication process has frequently been criticized for its tendency to prioritize immediacy over nuance, thus hindering the cultivation of informed judgment. However, acknowledging one’s ignorance can be the most scientifically accurate and ethically sound response. Scientific authority is not derived from omniscience, but rather from epistemic humility and a dedication to truth.

The proliferation of epidemiological indices, including the reproduction number (R_t_), case fatality rates, and vaccine efficacy metrics, has led to a state of information overload for the general public, frequently without adequate explanation or context. The issue at hand was not merely the availability of information, but rather the absence of clarity, contextualization, and methodological literacy in its communication. This phenomenon, which the World Health Organization has termed an “infodemic”, refers to the overabundance of information, both accurate and misleading, that complicates informed decision-making during a crisis [7].

In the context of vaccine hesitancy, it is becoming increasingly clear that purely rational approaches may not always be sufficient to change public attitudes. While scientific evidence and data are crucial in addressing public health concerns, there is a growing recognition that emotional and social factors play a significant role in shaping individual health decisions. Traditional science communication strategies, which often rely on presenting objective facts and figures, may not resonate with certain audiences, especially when these messages do not align with the values, beliefs, and emotional concerns of the public [8].

Persuasive communication strategies [9], often used in marketing and advertising, could offer a valuable complement to scientific communication. For example, the use of emotional appeals or the inclusion of trusted and relatable figures—such as celebrities or health advocates with “perfect physiques”—could be more effective in influencing public perceptions of vaccines and other health interventions. This approach exploits the emotional involvement of the public, which can sometimes prevail over logical reasoning in decision-making processes.

Moreover, the use of gamification and interactive digital tools [10] to engage individuals has also shown promise in fostering behavior change. Persuasive games that incorporate scientific messages can provide an engaging, interactive experience that may be more memorable and persuasive than static information or conventional public health campaigns. Although these approaches may raise ethical questions about manipulation, they underscore the need for science communicators to adapt their strategies to better connect with diverse audiences. This is particularly crucial when addressing health issues that require collective action, such as vaccination campaigns during pandemics. By integrating emotional, social, and interactive elements into science communication, it is possible not only to inform the public, but also to promote trust and engagement in ways that go beyond the mere dissemination of information.

This epistemic overload is not merely a temporary side effect of crises like COVID-19; rather, it signifies a profound structural transformation in the manner in which information functions within digital societies. Philosopher Byung-Chul Han [11] has conceptualized this transformation through the notion of infocracy—a regime in which power no longer depends on censorship or repression, but on the excess of information itself. In such systems, individuals are not silenced but rather disoriented, their cognitive attention fragmented by algorithmically curated content that prioritises emotional resonance over deliberative reasoning.

Han contrasts this with Michel Foucault’s model of the “disciplinary society”, in which social order was maintained through surveillance and institutional control. In the digital age, by contrast, control is exerted not by restricting access to information, but by saturating the informational environment, thereby diminishing citizens’ capacity for critical judgment. Algorithms, virality, and platform dynamics have replaced public debate with reactive attention, thereby rendering democratic deliberation increasingly fragile. From this perspective, infocracy can be regarded as the structural and political counterpart of the infodemic. While the infodemic denotes the spread of overwhelming or misleading information during crises, infocracy reveals how such chaos becomes systemically embedded in the logic of digital capitalism and governance. It reframes the problem: not simply a crisis of misinformation, but a mode of power that undermines both epistemic clarity and civic agency. This has direct implications for the communication of scientific knowledge, especially in the context of public health emergencies.

A structural driver of public confusion that merits greater attention is the interface between the scientific community and political decision-makers. In crises, the scientific community must present only robust evidence—or transparently label provisional hypotheses and advocate precautionary measures when certainty is unattainable. A cloud-based architecture that ensures equitable data access to both scientists and policymakers is essential to avoid the fragmentation of messaging. When scientific input fails to meet these epistemic and ethical standards—when hypotheses are expediently deployed as definitive facts—public trust erodes, and the infodemic’s impact is exacerbated by political instrumentalization of science [12].

Epidemiological indicators, such as R_t_ or the basic reproduction number (R_0_), are dynamic quantities that vary based on numerous factors, including population density, social behaviors, intervention measures, and even the type of diagnostic test used. In certain instances, policymakers have been observed to rely on such indicators without achieving a comprehensive understanding of their sensitivity or the confidence intervals that surround them. This has resulted in policy decisions that have been perceived by the public as arbitrary or inconsistent. Furthermore, pivotal epidemiological concepts, including herd immunity thresholds, secondary attack rates, and test positivity rates, were frequently distilled into simplistic slogans or oversimplified in media discourse, thereby compromising scientific integrity and eroding public trust.

Scientific knowledge, by its very nature, is provisional, probabilistic, and context-dependent. However, during the pandemic, scientific discourse was frequently erroneously equated with definitive truth. The pressure to provide unequivocal responses, in conjunction with rigorous media scrutiny and politicization, frequently resulted in the premature disclosure of findings, contradictory statements, and a decline in public confidence. The experience of the Green Pass implementation in Italy demonstrates how scientific policy tools can provoke societal polarization when communication fails to address public concerns and ethical implications [13]. In the case of WNV, although less visible than COVID-19, similar communication pitfalls have emerged. Despite the advances in diagnostics, entomological surveillance, and GIS-based risk mapping [14], public understanding of WNV transmission dynamics, zoonotic cycles, and preventive behaviours remains inadequate in practice. While awareness levels are generally high, a recent systematic review found that the actual adoption of protective behaviours – such as the use of insect repellents – varies significantly across populations and remains insufficient in several key groups, including older adults and pregnant women. Concerns regarding the safety of repellents, confusion about vector biology, and inconsistent risk perception further hinder the effectiveness of public health responses [15].

Trust in science must be fostered through the principles of clarity, integrity, and the acknowledgement of uncertainty. This process entails the discernment of expertise from opinion, and the promotion of an epistemic culture wherein the acknowledgement of limits is not perceived as a deficiency, but rather as a hallmark of credibility. The ethical foundation of scientific communication can be understood as encapsulated by the concept of “intelligent transparency”, described by the philosopher Onora O’Neill [16]. This term refers to a discourse that is explicitly committed to addressing uncertainties, evidentiary bases, and limitations. When these principles are disregarded by communicators, the potential exists for the transformation of science into a spectacle, susceptible to distortion.

Should the rational capacity to discern reality – and thus to seek truth – no longer be upheld as a foundational human faculty, then the inevitable consequence would be the rise of epistemic relativism. Within such a framework, science loses its normative authority, and the resulting erosion of truth engenders pervasive distrust, which, once consolidated, proves remarkably resistant to repair.

Concurrently, public perceptions of science are shaped by a combination of factors that extend beyond the realm of facts and institutional authority. Furthermore, these phenomena are impacted by social and psychological dynamics, including emotional framing, identity alignment, and value conflict [3, 4]. The credibility of scientific actors is contingent on their capacity to engage with diverse audiences, respond to epistemic pluralism, and communicate responsibly in environments characterized by political and cultural fragmentation.

This challenge is especially pronounced in the digital environment, where content disseminates expeditiously and the propagation of false information often supersedes factual accuracy. It has been demonstrated that social media platforms not only amplify misinformation but also the performance of expertise, where scientific authority is equated with media presence. In such a context, scientists must resist the lure of overexposure and instead practice what has been described as “epistemic responsibility“ [17], recognizing their role not only in generating knowledge but also in ensuring that this knowledge is communicated ethically and accessibly.

The ability to communicate effectively is insufficient in isolation; it must be accompanied by a thorough understanding of the subject matter and an ethical commitment to accuracy [18]. Scientists who are perceived as overly confident or detached risk alienating the public, even when their communication style is polished. Conversely, transparency regarding uncertainty, use of narrative, and empathetic engagement have been demonstrated to engender durable trust.

The ethical imperative extends beyond individual behavior to the structural relationship between science, media, and the public. In contemporary societies, expertise is frequently mediated by communication professionals, a process which can result in distortions if the intermediary lacks the requisite technical understanding. A central challenge, therefore, is to ensure that those who disseminate scientific findings are both scientifically literate and communicatively competent. Misalignments between scientists and communicators have repeatedly resulted in public confusion, particularly in instances where data are presented without contextual framing or is interpreted through ideological or commercial lenses.

This analysis also invites reflection on the concept of freedom in scientific expression. Authentic freedom in this domain does not lie in the liberty to articulate one’s thoughts and opinions, but rather the ethical responsibility to exercise discernment and precision in one’s communication, ensuring that statements are made with due care and responsibility. Charles Taylor [19] contends that freedom is not merely the absence of constraint, but rather the orientation towards that which is true and good. In this view, scientific freedom is defined as a disciplined commitment to truth-telling and resistance to the temptations of self-promotion, political expediency, and oversimplification. Hans Jonas [20] further posits that contemporary scientific pursuits must be complemented by an “ethic of responsibility”, particularly in domains that possess the potential to engender substantial harm. In the context of public health emergencies, it is imperative to exercise caution and ensure that scientific endeavors are not reduced to the pursuit of immediate, short-term objectives. Instead, there is a necessity to safeguard the integrity of scientific research, thereby safeguarding its long-term credibility.

It is imperative to acknowledge the pivotal role that young individuals, who have navigated the challenges of the pandemic during their formative years, play in shaping the future of science communication. The engagement of young people with digital tools, social media, and peer networks presents both opportunities and risks. Empowering them with scientific literacy, critical thinking, and ethical awareness is essential for fostering a new generation of communicators who can bridge the gap between expert knowledge and public understanding. Education systems and academic institutions must prioritize these competencies, not merely as technical skills but as civic virtues.

The recent management of WNV suggests that, despite the experiences of COVID-19, the structural conditions that generate infodemics persist. Recent studies have highlighted the persistence of fragmented information ecosystems, politicized scientific discourse, and inadequate public engagement mechanisms as key factors contributing to the proliferation of misinformation and the dilution of expert guidance [3, 4]. These structural dynamics, which include gaps in science communication training, institutional pressures, and the rapid mediation of information via digital platforms, contribute to the repeated failure to translate scientific knowledge into effective and trusted public health messaging. The recurrence of communication errors – unvetted interpretations, premature policy shifts, and inconsistent messaging – points to a failure to fully internalize the lessons of past emergencies. In order to avoid the perpetuation of cycles of mistrust, it is essential to reimagine science communication as an ethical practice, embedded in a broader framework of civic education and democratic responsibility.

In conclusion, the rebuilding of trust in science requires more than accurate data; it demands a transformation in how that data is interpreted, conveyed, and understood. It is incumbent upon scientific institutions to place communicative clarity at the pinnacle of their agenda, to promote epistemic humility, and to nurture a culture of accountability. Consequently, the public must be furnished with the instruments necessary to engage with scientific discourse in a critical manner. In periods of uncertainty, it is not infallibility that sustains trust, but rather integrity, competence, and a shared commitment to the public good.

## Data Availability

Not applicable.
